# The Levels of Biomarkers Interleukin 1 (IL-1) and Brain-Derived Neurotrophic Factor (BDNF) in Non-Invasive Conventional Rehabilitation and Robotic Rehabilitation Among Brain Injury Patients: A Narrative Review

**DOI:** 10.7759/cureus.68332

**Published:** 2024-08-31

**Authors:** Nur Ain Athirah Mohd Khairi, Muhammad Hafiz Hanafi, Nur Karyatee Kassim, Al Hafiz Ibrahim, Wan Muhamad Amir W Ahmad

**Affiliations:** 1 Department of Neuroscience, School of Medical Sciences, Universiti Sains Malaysia, Kota Bharu, MYS; 2 School of Dental Sciences, Universiti Sains Malaysia, Kota Bharu, MYS; 3 School of Medical Sciences, Universiti Sains Malaysia, Kota Bharu, MYS

**Keywords:** robotics, rehabilitation, interleukin-1, brain injuries, brain-derived neurotrophic factor

## Abstract

Acquired brain injury (ABI) is becoming increasingly common in Malaysia as a result of a rise in both strokes and accidents. The present review aims to explore the levels of serum inflammatory markers of interleukin-1 (IL-1) and brain-derived neurotrophic factor (BDNF) following conventional and robotic rehabilitation regimes among ABI patients and the association between serum biomarkers with the Medical Research Council (MRC) scale for muscle strength.

Online databases, namely ScienceDirect, PubMed, and Google Scholar were utilized by using search terms such as ‘Definition of brain injury’, ‘Epidemiology of brain injury’, ‘Interleukin-1 in stroke’, ‘BDNF in stroke’, ‘Interleukin-1 in traumatic brain injury’, ‘BDNF in traumatic brain injury’, ‘Interleukin-1 level and robotic rehabilitation’, ‘BDNF and robotic rehabilitation’, 'Interleukin-1 level and neurorehabilitation', and ‘BDNF and neurorehabilitation’. All types of articles with different levels of evidence were included along with other relevant review articles. Articles that were not in English and were not available in the full text were excluded.

The review identifies similar and no significant improvement in the treatment between conventional rehabilitation and robotic rehabilitation concerning serum biomarkers IL-1 and BDNF. This review also identifies that muscle strength and endurance training improved the level of serum BDNF in brain injury patients.

Therefore, this review provides evidence of the levels of IL-1 and BDNF in non-invasive conventional rehabilitation and robotic rehabilitation among brain injury patients, as well as their relation with the MRC scale, to give a good functional outcome that will enhance the quality of life of these groups of individuals.

## Introduction and background

Acquired brain injury (ABI) is a broad terminology that encompasses numerous etiologies that occur after birth, including vascular (stroke), traumatic brain injury (TBI), and anoxic injury, which are one of the main causes of impairment and a long-term serious public health problem [1]. Stroke is the second leading cause of death and the third leading cause of death and disability combined. Currently, three-quarters of global strokes are more likely to occur in low- and middle-income countries (LMICs) [2]. In 2014, the Centers for Disease Control and Prevention (CDC) reported that there were 2.53 million TBI-related emergencies, with 288,000 TBI-related hospitalizations and 56,800 TBI-related deaths in the United States [3]. Arulsamy et al. (2020) stated that TBI was the top three common admissions in Malaysia, and in 2009, nearly 80% of trauma cases were due to road traffic accidents [4]. The improvement in medicine and technology has led to an increase in survival rates. However, most survivors have after-effects that influence their ability to perform activities of daily living (ADLs) in a direct way. These effects can be sensorimotor, cognitive, emotional, or behavioral [1].

Since brain injury cases are high in Malaysia, rehabilitation is much more important to improve the quality of life. According to Arshad et al. (2022), early coordinated and multidisciplinary rehabilitation plays a major part in motor recovery after stroke [5]. However, based on our knowledge, the prognosis of the motor recovery of stroke survivors continues to be a major puzzle in the fundamental cellular mechanism, especially the changes in serum inflammatory markers (interleukins (IL)) and their interaction with neurotrophic biomolecules (brain-derived neurotrophic factor (BDNF)) of the central nervous system. Early periods of rehabilitation can optimize spontaneous neural plasticity and motor responsiveness, which results in maximized motor outcomes. However, traditional rehabilitation by a person is time-consuming and labor-demanding, which causes insufficient therapy dose, low engagement, and low motivation in the patient [5,6]. To give effective motor restoration depends on repetitive practice with high intensity [6]. In this new era, various technologies such as robotic rehabilitation have been developed that have shown promising results and are being used to promote repetitive task-specific training and concurrent feedback, as well as accurately measure functional improvement [5].

Since interleukin 1 (IL-1) acts as one of the earliest cytokines during the acute phase of neuroinflammation and BDNF is a key mediator for neuroplasticity, these two are the focus of this study [7,8]. This narrative review aims to explore the levels of serum inflammatory markers IL-1 and BDNF following conventional and robotic rehabilitation regimes among ABI patients. The association between the serum biomarkers and the Medical Research Council (MRC) scale for muscle strength is also explored. The evidence on the improvement of IL-1 and BDNF following robotic rehabilitation was hypothesized to give a good functional outcome that will enhance the quality of life of these groups of individuals.

## Review

Materials and methods

The electronic search was performed on databases, namely ScienceDirect, PubMed, and Google Scholar, by using search terms: ‘Definition of brain injury’, ‘Epidemiology of brain injury’, ‘Interleukin-1 in stroke’, ‘BDNF in stroke’, ‘Interleukin-1 in traumatic brain injury’, ‘BDNF in traumatic brain injury’, ‘Interleukin-1 level and robotic rehabilitation’, ‘BDNF and robotic rehabilitation’, 'Interleukin-1 level and neurorehabilitation', and ‘BDNF and neurorehabilitation’. All types of articles with different levels of evidence were included along with other relevant review articles. Articles that were not in English and were not available in the full text were excluded.

Brain injury

A stroke is an unexpected neurological condition caused by impaired blood vessel perfusion in the brain. Stroke can be classified as ischemic stroke, wherein insufficient blood and oxygen reach the brain causing thrombotic and embolic conditions, and hemorrhagic stroke, which is caused by bleeding or bursting blood vessels [9]. According to the Trial of Org 10172 in Acute Stroke Treatment (TOAST), the most prevalent subtype of ischemic stroke includes atherosclerosis, which affects the large artery, and embolism with cardiac genesis [10]. In thrombosis, there is an obstructive process caused by the build-up of plaque that prevents blood flow to some regions of the brain. An embolism occurs when there is a clot that is from another part of the body. A clot at the valve or chamber of the heart causes a reduction of blood toward the brain. Severe stress and necrosis are brought on by this embolism. Necrosis results in disruption of the plasma membrane, enlargement of the organelles that leak cellular contents into the extracellular space, and loss of neuronal function [9,11]. 

Hemorrhagic stroke is usually caused by hypertension due to bleeding into the brain from the rupture of blood vessels and can be further divided into subarachnoid hemorrhage (SAH) and intracerebral hemorrhage (ICH) [12]. In ICH, blood clots in the blood vessels cause an abnormal build-up of blood in the brain. Hypertension, abnormal vasculature, overuse of anticoagulants, and thrombolytic agents are the primary causes of ICH. When there is a head injury or cerebral aneurysm, blood builds up in the brain's subarachnoid space, resulting in subarachnoid hemorrhage [9]. 

Common post-stroke disabilities include motor impairments like central facial paresis, hemiplegia, and hemiparesis (left or right side weakness or paralysis). Language and speech disorders are also common. Other disabilities include altered states of consciousness, reduced blood flow to particular areas of the brain, and impaired vision. Each of these disabilities has a substantial impact on the quality of life for stroke patients [13]. Nearly all patients require rehabilitation (physiotherapy, occupational therapy, and speech therapy) based on their needs [14].

Imaging modalities are used in suspected stroke cases to accurately diagnose the condition and guide treatment decisions. Identifying the kind, location, and severity of a stroke allows for prompt intervention. Initially, non-contrast computed tomography (CT) is used to rule out stroke caused by hemorrhage. Diffusion-weighted imaging (DWI) in conjunction with magnetic resonance imaging (MRI) is a highly sensitive technique that can detect early ischemic changes and locate the infarcted brain tissue. By identifying obstructed blood vessels, CT angiography offers comprehensive details about the cerebral vasculature [15].

Further, TBI results from a blow, bump, or jolt to the head, or from a penetrating head injury and blast overpressure exposure such as in military combat that causes disruptions to normal brain function. These disruptions alter cerebral blood flow and brain metabolism, leading to neurological impairments and cell death similar to cellular mechanisms in stroke [3,16]. Injuries can be separated into two categories. Primary injury occurs during trauma. The two types of primary injuries are focal and diffuse brain injuries. Research indicates that patients with moderate to severe TBI (based on the Glasgow coma scale (GCS)), post-traumatic amnesia, and loss of consciousness duration experience both types of injuries. Necrotic areas of neuronal and glial cells concentrated at the coup with impaired blood supply cause hematomas, epidural, subdural, and intracerebral hemorrhages to occur at specific brain layers. When the brain rebounds and strikes the skull, tissues opposite or surrounding the coup (contrecoup) may sustain a secondary contusion as a result of a secondary impact. The mechanism in primary injuries results in delayed and prolonged secondary injuries. The effects of primary injuries can cause additional cellular damage, which is the cause of secondary types of TBI. The secondary injuries may appear hours or days later. Secondary injuries can occur due to various factors such as excitotoxicity, mitochondrial dysfunction, oxidative stress, lipid peroxidation, neuroinflammation, axon degeneration, and apoptotic cell death [17,18]. In particular, clinicals of injuries with GCS scores less than 13 use MRIs or CT scans of the head. When it comes to minor injuries, an MRI or CT scan should only be used when the patient's GCS score falls between 13 and 15. An MRI may be considered if there are ongoing symptoms or discrepancies between the CT and clinical findings, such as poor neurologic status and negative CT results [19].

Similar to stroke, TBI can result in disability based on the extent of brain damage. TBI patients may exhibit verbal and cognitive deficits, physical impairment arising from orthopedic injuries sustained in an accident, and neuromotor impairments directly caused by upper motor neuron lesions. Physical impairment after TBI can cause patients to experience chronic health problems. TBI comorbidities are associated with increased mortality rates and secondary impairment arises from prolonged bed rest and immobilization. Therefore, physiotherapists ought to support and motivate their patients to identify and engage in their preferred physical activities [20]. 

Conventional and robotic rehabilitation

The goal of neurorehabilitation is to prevent additional losses, repair damage, restore function using novel techniques, and prevent the body from adjusting to functional deficits brought on by neurological disease or injury [21]. In this new era, several interventions for technology-based stroke rehabilitation have given good results in improving patients’ functional mobility and independence. Technologies such as robotic-assisted systems, virtual and augmented reality, telerehabilitation, exergames wearable sensors, and smartphone applications are used to promote repetitive task-specific training, active participation of patients, integrating constructive and concurrent feedback, and accurately measuring functional improvement [5].

In conventional rehabilitation, Xing and Bai (2020) stated that running exercise can improve neurotrophic factors and other modulators linked to synaptic plasticity, which may help in learning and attention during the initial phases of stroke rehabilitation [22]. Another study was done by Linder et al. (2019) on conventional rehabilitation to determine the differential effects of forced or voluntary aerobic exercise in conjunction with upper-extremity repetitive task practice on the recovery of motor function in adults with stroke. They found that forced exercise performed prior to repetitive task practice improved motor skills more than voluntary exercise or stroke-related education. To enhance motor recovery after a stroke, aerobic and forced exercise should be taken into consideration [23].

The study on combining virtual reality with conventional rehabilitation was done by Rodríguez-Hernández et al. (2023) to improve post-stroke hand motor function. They found that conventional rehabilitation combined with a specific virtual reality technology system can be more effective than conventional rehabilitation alone in enhancing hand motor function and voluntary movement in muscle tone for subacute post-stroke patients [24]. Sramka et al. (2020) conducted a study to confirm the viability of combining virtual reality with traditional methods for stroke patients’ rehabilitation and found that patients who underwent combined rehabilitation had the potential to accelerate rehabilitation and increase motivation toward selected groups of patients after stroke [25]. According to the Malaysia Ministry of Health (MOH), the primary goals of physiotherapy for conventional stroke rehabilitation are to accelerate the neuromuscular system's recovery, including voluntary limb movement, balance, coordination, and walking ability; to enhance functional activities; to guarantee optimal mobility and independence; and to lower complications. However, Malaysia still faces difficulties in offering patients the best possible rehabilitation care. Using robotic rehabilitation can improve the treatment of conventional therapy. Robotics can be used to provide precise and reliable treatment. Anywise, conventional rehabilitation remains irreplaceable because it can provide precise movement in therapy while assisting with what the patients need and enhancing patient outcomes [26]. Table 1 shows the summary of the selected studies in conventional and robotic rehabilitation.

**Table 1:** Summary of studies on conventional and robotic rehabilitation RTP: Repetitive task practice; AE: Aerobic exercise

No	Authors	Study Design	Objectives	Outcome/Conclusions
1.	Xing and Bai (2020) [22]	A narrative review	To discuss the mechanisms by which exercise-induced neuroplasticity improves motor function and cognitive ability post-ischemic stroke.	Exercise that involves running has been shown to increase neurotrophic factors and other synaptic plasticity modulators, which may help in learning and attention in the early phases of stroke recovery.
2.	Linder et al. (2019) [23]	This report is a secondary analysis of data from two randomized clinical trials	To determine the differential effects of upper-extremity RTP and forced or voluntary AE on motor recovery function in adults with stroke.	Forced exercise administered prior to repetitive task practice improved motor skills more than voluntary exercise or stroke-related education.
3.	Sramka et al. (2020) [25]	Intervention study	To verify the viability of combining virtual reality with traditional methods for stroke patients’ rehabilitation.	Patients who underwent combined rehabilitation have the potential to accelerate rehabilitation and increase motivation toward selected groups of patients after stroke.
4.	Nik Ramli et al. (2021) [26]	A systematic review	The efficiency of robot-assisted physical therapy, especially in terms of enhancing motor abilities in stroke patients in Malaysia.	Robotic rehabilitation can help enhance treatment from conventional therapy. Robotics treatment is precise and consistent.

Biomarkers in brain injury

Based on research, the latest biomarkers in brain injury recovery are S100B, glial fibrillary acidic protein (GFAP), neuron-specific enolase (NSE), tau protein, micro RNA, BDNF, and IL-1 [27-29]. As mentioned, IL-1 acts as one of the earliest cytokines during the acute phase of neuroinflammation and BDNF is a key mediator for neuroplasticity; these two are the focus of this study [7,8].

IL-1

Cytokines are released into the peripheral blood and injured cerebral tissue following brain inflammation [10]. In the process of cell activation, differentiation, and proliferation, cytokines play a significant role in immune system modulation. These substances function as pro-inflammatory mediators, releasing the primary inflammatory factors, including IL-1. After a TBI, IL-1β is crucial for immune system regulation and the development of secondary injuries [10,30].

A systematic review and meta-analysis was done by Mavroudis et al. (2024) on inflammatory biomarkers in mild traumatic brain injury (mTBI). They stated that IL-6, tumor necrosis factor alpha (TNFα), and interleukin-1 beta (IL-1β) plasma levels increase during the development of early post-concussion symptoms [31]. Malik et al. (2023) reported eight studies comparing the blood levels of IL-1β between mTBI patients and healthy controls; three of those studies demonstrated a significant increase in mTBI patients’ levels of IL-1β compared to healthy controls, at least by one point. One study, however, demonstrated a significant decrease in IL-1β levels in the mTBI group when compared to the health control group, and the other four studies found no discernible blood differences between those two groups. We can see from this experiment that there is still uncertainty regarding the mechanism of IL-1 because of the various outcomes [32]. Limited studies give strength to future studies to conduct it. 

Based on our knowledge, no studies have demonstrated IL-1’s connection to rehabilitation. However, one study conducted by Docherty et al. (2022) reported that interleukin 6 (IL-6) is involved in the propagation of a pro-inflammatory state and attracts neutrophils to the site of injury. The level of IL-6 rises with increased exercise volume, intensity, and duration, as well as individual fitness capacity. This study also found that after exercise, levels of interleukin 10 (IL-10) and IL-1 increase. These two molecules are known to play a significant role in immune regulation and improve the body's natural defenses against inflammation. This study helps us to understand that increasing exercise may result in more muscle inflammation and a faster healing process [33]. Table 2 shows the summary of the studies on IL-1 in brain injury and neurorehabilitation. Future studies about the relationship between IL-1 and rehabilitation are important to provide a good and effective intervention for patients based on evidence. 

**Table 2:** Summary of review articles on interleukin-1 in brain injury and neurorehabilitation mTBI: Mild traumatic brain injury; IL-6: Interleukin 6; TNFα: Tumor necrosis factor alpha; IL-1β: Interleukin 1 beta

No	Authors	Study Design	Objectives	Outcome/Conclusions
1.	Mavroudis et al. (2024) [31]	A systematic review and meta-analysis	A summary of the current knowledge on the role of inflammation in the pathogenesis of mTBI and the potential of some inflammatory biomolecules as biomarkers of mTBI.	IL-6, TNFα, and IL-1β plasma levels increase during the development of early post-concussion symptoms.
2.	Malik et al. (2023) [32]	A systematic review and meta-analysis	To synthesize data related to levels of inflammatory cytokines in patients with mTBI.	Eight studies comparing the levels of IL-1β in blood between mTBI patients and healthy controls: three studies showed a significant increase in the level of IL-1β in mTBI compared with healthy controls by a minimum of one point; one of the studies showed significantly reduced IL-1β levels in mTBI compared to healthy control groups; four studies found no significant difference in blood between the two groups.
3.	Docherty et al. (2022) [33]	Narrative review	A summary of the existing literature on the relationship between exercise and the immune system, focusing on the ways that exercise-induced cytokine expression affects immune response and inflammation.	The immune system experiences physiological changes from exercise. Significant increase in IL-6 derived from muscle, involved in the coordination of an anti-inflammatory immune response during exercise. IL-6 is also a pro-inflammatory cytokine.

BDNF

Neuroplasticity, which is involved during brain injury recovery, is the capacity of neural circuits to adapt both structurally and functionally, where the brain adapts its structure and function [34,35]. BDNF plays an important role in TBI restorative processes, including synaptogenesis, axonal sprouting, and neuronal survival [36]. 

Yin et al. (2020) conducted a study on the effects of BDNF on inhibiting neuroinflammation and promoting neurological recovery by using BDNF fused with a collagen-binding domain (CBD-BDNF). In the study, the regulatory effects of BDNF and CBD-BDNF microglia’s inflammatory response were studied in a TBI mice model in vivo and lipopolysaccharide (LPS)-stimulated microglia experiment in vitro. BDNF fused with a collagen-binding sphere was used to maintain a sufficient concentration of BDNF in the TBI hemisphere. The study showed that BDNF and CBD-BDNF both had equal effects on reducing pro-inflammatory response while enhancing the anti-inflammatory response that LPS induced in vitro in microglia even though BDNF was shown to be much weaker than CBD-BDNF [37]. Based on the study, whether BDNF was fused with a collagen-binding domain or not, both have effects on reducing pro-inflammatory reactions. This study gives evidence that BDNF promotes brain injury recovery.

Studies on the association between the level of BDNF and neurorehabilitation have been done recently. Chen et al. (2023) in their study on the biomarkers associated with functional improvement after stroke rehabilitation showed that there was a significant increase in the level of BDNF, and this change was also linked to function improvement. They also reported that low serum BDNF levels were significantly associated with poor functional outcomes and high mortality [38]. Koroleva et al. (2020) did a study where they divided patients into three groups. One group received motion sensors and augmented reality-assisted classical rehabilitation as well as motor rehabilitation, the second group only received classical rehabilitation, and the final group received no rehabilitation treatment. The results showed that BDNF levels significantly increased during the phase with augmented rehabilitation. Those groups who received rehabilitation had similar BDNF levels, while untreated patients had significantly low levels. However, they concluded that BDNF does not have a relation to motor improvement but may react to active treatment [39]. According to Niimi et al. (2016), low-frequency repetitive transcranial magnetic stimulation (rTMS) combined with rehabilitation can activate BDNF and improve motor function in the affected limbs. They also reported that an increase in the number of rehabilitation results in an increase in serum BDNF concentration [40]. Luo et al. (2019) reported that aerobic exercise for four weeks may increase the level of mature BDNF (mBDNF)/pro-BDNF (proBDNF) in the ischemic hippocampus of rats [41]. Xing and Bai (2020) reported that, in an animal experiment, promoting brain plasticity after ischemic, low-volume, high-intensity training (HIT) may be more effective than moderate-intensity training (MIT) due to more effective motor function improvement, which may be possible from an increased ratio of mBDNF/proBDNF in the hippocampus. For robotic rehabilitation, they summarized that it was effective in improving upper limb motor function, learning, and memory in stroke patients, and the possible mechanism involves improving neuroplasticity [22]. The summary of the studies on BDNF in brain injury recovery and neurorehabilitation is shown in Table 3.

**Table 3:** Summary of studies on BDNF in brain injury recovery and neurorehabilitation TrkB: Tropomyosin receptor kinase B; BDNF: Brain-derived neurotrophic factor; CBD-BDNF: BDNF fused with a collagen-binding domain; SOD: Superoxide dismutase; ALB: Albumin; HB: Hemoglobin; CAT: Catalase; NE: Norepinephrine; ET: Endothelin; LPS: Lipopolysaccharide; rTMS: Repetitive transcranial magnetic stimulation; MMP-9: matrix metalloproteinase 9; mBDNF: Mature BDNF; proBDNF: Pro-BDNF; HIT: High-intensity training; MIT: Moderate-intensity training

No	Authors	Study Design	Objectives	Outcome/Conclusions
1	Yin et al. (2020) [37]	Intervention study	To explore the potential of CBD-BDNF in regulating inflammatory response and promoting neurological recovery in traumatic brain injury, particularly through the TrkB signaling pathway.	BDNF and CBD-BDNF both had equal effects on reducing pro-inflammatory response while enhancing the anti-inflammatory responses that LPS induced in vitro in microglia even though BDNF was shown to be much weaker than CBD-BDNF.
2	Chen et al. (2023) [38]	A systematic review and meta-analysis of randomized controlled trials	To identify blood and cerebrospinal fluid biomarkers that are correlated to functional improvement of stroke patients after rehabilitation therapy, and provide ideas for the treatment and evaluation of stroke patients.	The concentration of serum BNDF, NE, ET, and glutamate, and peripheral blood SOD, ALB, HB, and CAT suggest the function improvement of stroke patients.
3	Koroleva et al. (2020) [39]	Intervention studies	To clarify the potential function of BDNF in the early motor training-assisted recovery from ischemic stroke.	Although they appear to respond to active treatment, BDNF level variations are not always associated with improvements in motor function. Without active rehabilitation treatment, BDNF tends to decrease.
4	Niimi et al. (2016) [40]	Intervention studies	To examine the molecular effects of rTMS on serum levels of BDNF, its precursor proBDNF, and MMP-9 in post-stroke patients with upper limb hemiparesis.	Through the activation of BDNF processing, the combination therapy of rehabilitation and low-frequency rTMS appears to improve motor function in the affected limb. ProBDNF, the precursor of BDNF, and BDNF itself may be useful biomarkers for post-stroke motor recovery.
5	Luo et al. (2019) [41]	A preclinical experimental study	To investigate the role of BDNF and proBDNF in the development of depression behavior in post-stroke depression rats and evaluate the effects of aerobic exercise on the regulation of BDNF isoforms	Aerobic exercise for four weeks may increase the level of mBDNF/proBDNF in the ischemic hippocampus of rats.
6	Xing and Bai (2020) [22]	A narrative review of studies	To discuss the mechanisms by which exercise-induced neuroplasticity improves motor function and cognitive ability post-ischemic stroke.	Promoting brain plasticity after ischemic, low volume of HIT may surpass MIT from the effectiveness of motor function improvement through an increased ratio of mBDNF/proBDNF in the hippocampus. In robotic rehabilitation, it is effective in improving upper limb motor function, learning, and memory in stroke patients, and the possible mechanism involves improving neuroplasticity.

MRC scale

The MRC scale for muscle strength is a valid and trustworthy assessment tool used to track a patient's progress and is essential in choosing the right treatments. Typically, stroke patients’ muscle loss is assessed using this scale. Hemiparesis, which is associated with muscle weakness and the inability to exert enough muscle force to complete a task, is a condition that occurs after brain damage [42]. 

Based on our knowledge, there are limited studies on MRC that compare conventional and robotic therapy. Major et al. (2021) examined robotic versus classical physical therapy pre- and post-treatment for neurological diseases by measuring muscle strength and range of motion. Clinical measures that were used were the MRC scale, goniometry, and dynamometry. They conducted tests in the upper limb using both robotic and traditional physical therapy, both before and after rehabilitation, for the same amount of time. However, the MRC scale showed no significant difference between both therapies [43]. Singh et al. (2021) conducted a study among stroke patients to compare the rehabilitation effectiveness of robotic therapy training and conventional therapy using the modified Ashworth scale (MAS), active range of motion (AROM), Barthel index, Brunnstrom stage, and Fugl-Mayer (FM) as clinical scales. The results showed that both treatments had an improvement in all clinical outcomes except MAS. Even though both gave improvement, the group with robotic therapy training showed significantly higher improvement than the group with conventional therapy [44]. Taravati et al. (2022) studied the impact of adding robotic rehabilitation with conventional rehabilitation therapy to improve the quality of life, motor function, cognition, and emotional status of a hemiplegic patient with stroke. The results showed both groups, only conventional therapy and conventional therapy combined with robotic therapy, had improvements, but they were not quite significant. They concluded that robotic rehabilitation is an effective and valuable addition to traditional neurological rehabilitation in the treatment of stroke patients. It offers a favorable alternative with minor benefits and is also advantageous in terms of work capacity and psychological recovery [45]. Arazi et al. (2021) did a study on the effectiveness of strength and endurance exercise on brain neurological factors in older men wherein they reported that both strength and endurance interventions are effective in elevating BDNF, insulin-like growth factor 1 (IGF-1), and platelets, without any significant difference between them [46]. The summary of studies on the MRC scale's relation with conventional and robotic rehabilitation and serum biomarkers is shown in Table 4.

**Table 4:** Summary of studies on the MRC scale's relation with conventional and robotic rehabilitation and serum biomarkers MRC: Medical Research Council; BDNF: Brain-derived neurotrophic factor; IGF-1: Insulin-like growth factor 1

No	Authors	Study Design	Objectives	Outcome/Conclusions
1.	Major et al. (2021) [43]	Comparative study	To compare the effectiveness of robotic-assisted rehabilitation methods with manual physical rehabilitation therapy for upper-limb medical recovery in patients suffering from motor deficits caused by various neurological diseases.	The MRC scale showed no significant difference between both therapies.
2.	Singh et al. (2021) [44]	A randomized controlled trial	To assess the efficacy of robotic therapy training sessions in stroke patients' rehabilitation using both clinical scales and neurophysiological measures in comparison to dose-matched conventional therapy.	Both give improvement, those groups with robotic-therapy training seem to show significantly higher improvement than those with conventional therapy.
3.	Taravati et al. (2022) [45]	A randomized controlled study	To determine whether adding robotic therapy to a traditional rehabilitation program has an impact on hemiplegic patients' quality of life, motor function, cognition, and emotional state.	Robotic rehabilitation complements traditional neurological rehabilitation for stroke patients, offering minor benefits and advantages in work capacity and psychological recovery.
4.	Arazi et al. (2021) [46]	Intervention study	To test the effects of strength and endurance exercise on brain neurobiological factors in older men.	Following exercise, serum BDNF, IGF-1, and platelet concentrations significantly increased in both the endurance and strength groups compared to the control group. Following exercise, no statistically significant differences were found between the endurance and strength groups.

Summary

Evidence suggests an increasing number of brain injury cases in Malaysia. Due to brain injury, patients have disabilities that cause reduced quality of life. Physiotherapy is one of the effective treatments to improve function and daily living. Since traditional rehabilitation by a person is time-consuming and labor-demanding, robotic-assisted therapy has been developed that has shown a promising result in promoting repetitive task-specific training and concurrent feedback, as well as accurately measuring functional improvement. However, limited studies have been found on IL-1 in rehabilitation and BDNF in rehabilitation for both conventional rehabilitation and robotic rehabilitation regimes. The MRC scale is also important to describe the improvement of muscle strength in patients. Yet, limited studies on the MRC scale were found concerning conventional rehabilitation and robotic rehabilitation regimes. The limitation of this review is a lack of recent original articles, as this represents a promising research gap that needs to be filled. Future studies need to be related to IL-1, BDNF, and MRC scale in conventional and robotic rehabilitation regimes to strengthen insights into this area and provide a good, effective treatment with proof of evidence-based.

**Figure 1:**
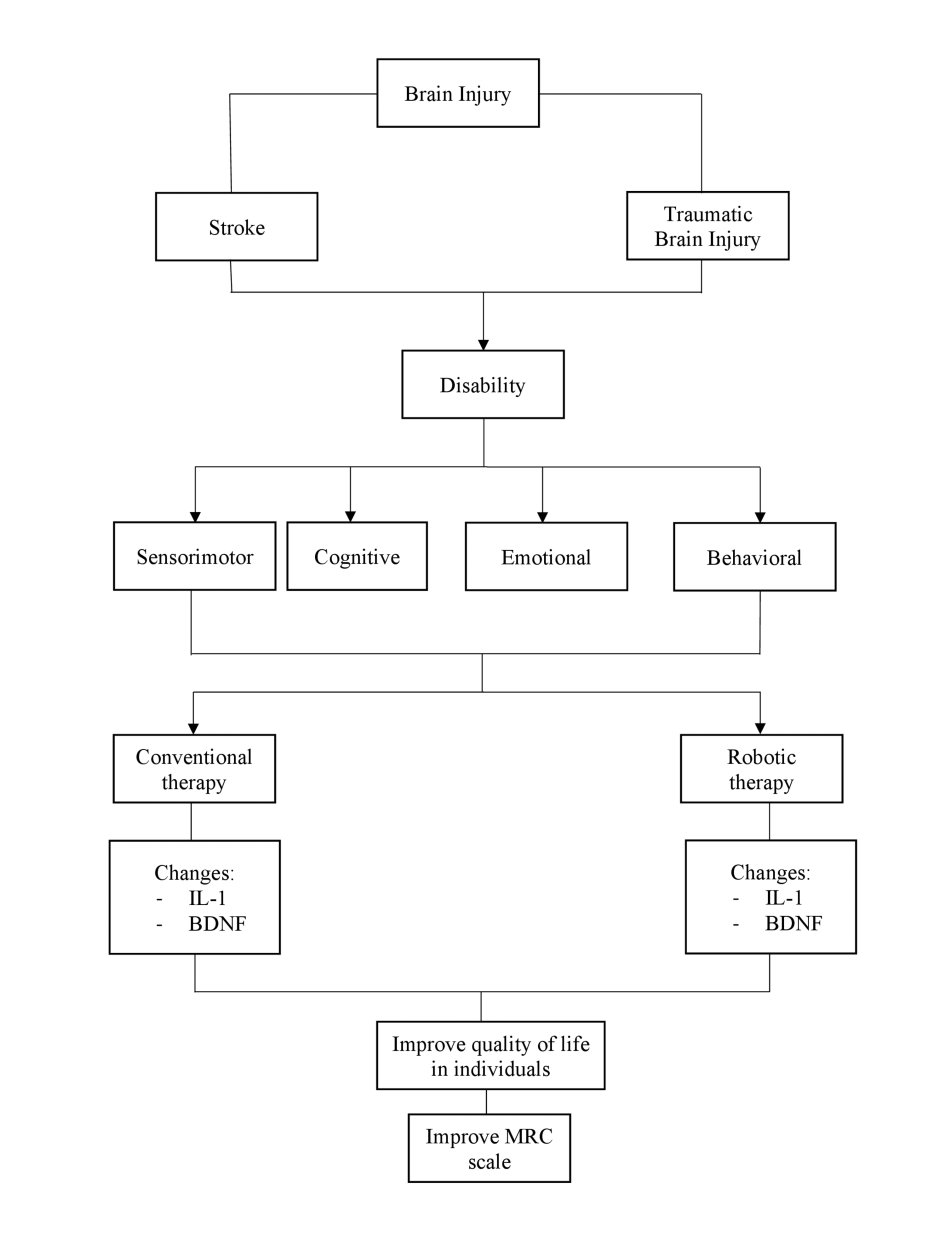
Outline of the key concepts in this narrative review MRC: Medical Research Council; BDNF: Brain-derived neurotrophic factor; IL-1: Interleukin 1

An outline of the key concepts discussed above is presented in Figure 1.

## Conclusions

The levels of serum inflammatory markers IL-1 and BDNF following conventional and robotic rehabilitation regimes among patients with ABI exposure seem to give uncertainty in the result. Certain evidence reported similar effectiveness of treatment and other studies with robotic assistance reported a higher result. Studies on the relation between the MRC scale and serum biomarkers IL-1 and BDNF are also limited. However, one study suggested that with muscle strength and endurance training, there is an improvement in BDNF. Future studies are recommended to be conducted on the relation of biomarkers IL-1 and BDNF with conventional and robotic rehabilitation, as well as with the MRC scale, to give a good functional outcome that may improve the quality of life of these groups of individuals.
